# The Idiom Processing Advantage is Explained By Surprisal

**DOI:** 10.1111/cogs.70085

**Published:** 2025-07-20

**Authors:** Michaela Socolof, Timothy J. O'Donnell, Michael Wagner

**Affiliations:** ^1^ Department of Brain and Cognitive Sciences Massachusetts Institute of Technology; ^2^ Department of Linguistics McGill University

**Keywords:** Idioms, Processing, Language production, Language comprehension, Surprisal, Compositionality

## Abstract

It has been repeatedly found that idioms are processed faster than syntactically matched literal phrases, in both comprehension and production. This has led to debate about whether idioms are accessed as chunks or built compositionally, with different studies attempting to measure the effect of compositionality on processing, with differing conclusions. This paper looks at idiom processing through the lens of information update, in particular *surprisal theory*, which is a standard theory of sentence processing. Compositionality is just one aspect of a word's predictability; we argue that surprisal, as an expectation‐based theory, provides a more general unifying framework for understanding the idiom processing advantage. In this paper, comprehension and production experiments on verb‐object idioms reveal that the idiom processing advantage can be largely explained by the fact that idioms have lower surprisal than matched literal phrases. The results indicate that the idiom advantage manifests primarily on the noun in verb‐object idioms.

## 1 Introduction

Within the literature on sentence processing, it has been consistently and robustly found that idiomatic phrases are quicker to process than literal counterparts (e.g., Carrol & Conklin, 2020; Swinney & Cutler, 1979; Siyanova‐Chanturia & Lin, 2018; Titone, Lovseth, Kasparian, & Tiv, 2019; Van Lancker, Canter, & Terbeek, 1981). A literature has grown around seeking to explain this difference, known as the *idiom processing advantage*, with studies investigating the effects of various combinations of predictors. While the specific predictors and how they are determined vary, most studies have found that some notion of familiarity or recognizability helps account for the quicker processing speed of idioms. These studies have found that idioms, by virtue of being highly familiar sequences of words, are retrieved faster and more easily than less familiar, or entirely unfamiliar, phrases. The familiarity effect has been used in some work to support a *direct retrieval* theory of idiom processing, which holds that idioms are accessed directly from the lexicon, like words, and that this proceeds faster than the act of composing multiple words, as one must do with a literally interpreted phrase (Bobrow & Bell, 1973; Cacciari & Tabossi, 1988; Swinney & Cutler, 1979). From a theoretical perspective, this accords with theories that treat idioms as nondecomposable lexical items (e.g., Bobrow & Bell, 1973; Swinney & Cutler, 1979; Weinreich, 1969).

A second potential factor in explaining the idiom processing advantage has to do with the relationship between the meanings of the individual words in a phrase and the meaning of the phrase as a whole. Idioms have often been described as noncompositional—unlike in typical language use, the meanings of the words in an idiom do not combine according to predictable and systematic rules to yield the meaning of the phrase. For example, knowing the meanings of *spill* and *beans* is not enough to determine that *spill the beans* means something like “reveal a secret” (e.g., Bobrow & Bell, 1973; Portner, 2005; Radford, 2004; Swinney & Cutler, 1979; Weinreich, 1969). Therefore, some recent work has argued that meaning composition must play a role in the idiom advantage, based on experiments showing that idiom processing may be sensitive to the transparency of the mapping between the idiom's meaning and the meanings of its component words, though whether such an effect is real and if so, the precise nature of it, remains contested (e.g., Gibbs, Nayak, & Cutting, 1989; Titone & Connine, 1999; Titone & Libben, 2014). In our view, there are two likely reasons for these mixed results. First, studies investigating the role of compositionality on idiom processing make the assumption that processing time will be sensitive to differences in compositionality, when this is not in fact guaranteed. Second, there is no generally accepted way of measuring how compositional a phrases is, which has led to a variety of related but distinct measures in the literature, many of which end up targeting different phenomena. We expand on both of these issues below, and the studies reported in this paper address both, in an effort to shed light on the reasons behind the idiom advantage.

We begin by investigating the assumption that processing time is sensitive to differences in compositionality. In particular, we consider an alternative type of theory about the factors affecting the processing time of words in context; the most well‐known theory of this type is *surprisal theory* (Hale, 2001; Levy, 2008; Smith & Levy, 2013; Wilcox, Pimentel, Meister, Cotterell, & Levy, 2023), which hypothesizes that processing time reflects how expected a word is in context, with unexpected words causing greater processing difficulty. The processing difficulty of a word $w_{i}$ is measured in *surprisal*, which is the negative log probability of the word given its context c. This quantity measures the information update associated with the word in context.

(1) $$\begin{array}{c} \begin{array}{c} s\left(w_{i}\right)=-\log_{2}p\left(w_{i}|c\right). \end{array} \end{array}$$

Previous studies of idiom processing have generally not investigated the role of surprisal in explaining the idiom processing advantage. We believe this is a significant oversight, as surprisal theory provides an overarching framework for capturing processing difficulty. Furthermore, surprisal theory has been argued to be a *causal bottleneck* between linguistic representations and processing difficulty (Levy, 2008). In other words, surprisal subsumes various linguistic factors, and it is the surprisal quantity, rather than any of those individual factors, that is reflected in processing time. Under this framework, compositionality would be merely one of many factors that goes into a word's expectedness, and the surprisal effect would capture any compositionality effect. The causal bottleneck claim is a stronger claim than standard surprisal theory, and there is some evidence showing that surprisal is not in fact a bottleneck for all possible factors affecting processing (Staub, 2024). We believe that the conflicting findings from previous work on idiom processing can be better understood if we look at them through the lens of a standard theory of processing, and we investigate whether differences in surprisal between idioms and nonidioms can account for the idiom processing advantage. In order to test this hypothesis, we include in our models of processing time a fine‐grained compositionality predictor that measures the relationship between the meaning of a phrase and the meanings of its pieces, and we find that suprisal largely, but not entirely, explains the idiom processing advantage, contributing to evidence against the causal bottleneck theory. We motivate the specific choice of compositionality measure in Section 3.

The bulk of the literature on idiom processing has focused on comprehension, where the idiom processing advantage has been consistently found (e.g., Carrol and Conklin, 2020; Swinney & Cutler, 1979; Titone et al., 2019). There has been less work looking at idiom production, with the few studies that have been done indicating that idioms are faster in production as well (Lovseth, de la Parra, Wagner, & Titone, 2011; Siyanova‐Chanturia & Lin, 2018; Van Lancker et al., 1981). We carry out both a comprehension and a planned production study on idioms versus nonidioms, investigating whether a compositionality measure has any additional effect beyond that of surprisal in explaining differences in spoken duration and/or reaction time (RT). We find evidence of the idiom processing advantage in both studies, and we further find that the idiom processing advantage is primarily explained by surprisal, yet also exhibits a compositionality effect in the production experiment. The results of our production study are particularly interesting, as a planned production task crucially differs from a comprehension task in that participants know in advance the sentence they will be saying, and, therefore, their speaking time may not straightforwardly reflect their reaction to seeing a particular phrase. These results have implications for theories of idiom processing, the causal bottleneck principle of surprisal theory, and the relationship between comprehension and production.

## 2 Relation to previous work

The majority of studies on idioms in psycholinguistics have looked at comprehension as a window into processing, and these studies have overwhelmingly found that idioms are processed more quickly than literal phrase controls (e.g., Carrol and Conklin, 2020; Swinney & Cutler, 1979; Titone et al., 2019).^1^ Furthermore, there is consensus that the familiarity or recognizability of a phrase, though measured differently across different studies, helps explain the idiom processing advantage, given that idioms tend to be familiar phrases (Abel, 2003; Cacciari & Tabossi, 1988; Carrol and Conklin, 2020; Cronk & Schweigert, 1992; Carrol, Littlemore, & Dowens, 2018; Cronk, Lima, & Schweigert, 1993; Libben & Titone, 2008; Schweigert, 1986; Tabossi, Fanari, & Wolf, 2009; Titone et al., 2019). This family of predictors includes measures such as *final‐word predictability*—a measure of how likely people were, under experimental conditions, to predict the final word of an idiom given its beginning—and *familiarity*—a behavioral measure of how many people recognize a particular idiom. These measures capture the idea that when one encounters the beginning of an idiom, one is primed to expect a particular continuation. The measures are thus highly related to the notion of surprisal, so it is perhaps unsurprising that they have been found to predict processing time, though in previous work, these measures have been used to give a single value per phrase, whereas our study considers surprisal values of individual words within a phrase. Our study takes seriously the ideas of surprisal theory and uses surprisal values as a standardized measure of how much one expects that an utterance will continue in a certain way. The only other study we are aware of that investigates surprisal in this domain is Rambelli, Chersoni, Senaldi, Blache, and Lenci (2023), which looks at whether compositional‐but‐frequent collocations pattern more like idioms or more like regular compositional phrases during comprehension. The authors find that collocations pattern similarly to idioms in both self‐paced reading times and surprisal values.

As for predictors related to compositionality, the most common measure in the literature is *decomposability*, which is typically drawn from experimental ratings of the extent to which the words in an idiom contribute to the idiomatic meaning, though there is no standard procedure for obtaining these ratings. As an example to illustrate the notion of decomposability, Gibbs et al. (1989) describe how “the phrase *fall off the wagon* is less decomposable than *pop the question* because the meaning that *fall* contributes to *fall off the wagon* is not as salient as the meaning that *pop* contributes to *pop the question*.” There have been conflicting results regarding the effect of decomposability on idiom processing, with some studies finding a facilitative effect of decomposability (Caillies & Butcher, 2007; Gibbs et al., 1989), and others finding no such effect (Titone & Connine, 1999; Tabossi, Fanari, & Wolf, 2008; Tabossi et al., 2009; Tabossi, Wolf, & Koterle, 2009; Titone & Libben, 2014; Titone et al., 2019). Libben and Titone (2008) found a facilitative effect for offline comprehension measures (i.e., ratings and judgments), but not for an online self‐paced moving window measure. Carrol and Conklin (2020) actually found that decomposability *increases* reading time. Familiarity and decomposability have also been found to interact, with Titone et al. (2019) reporting that familiarity only facilitated reading time for phrases that had low decomposability, and Libben and Titone (2008) reporting that decomposability has a greater effect for less familiar phrases. A few studies have attempted to disentangle these results. The study reported in Titone et al. (2019), which initially looked at the effects of familiarity and decomposability on verb‐object idioms using eye‐tracking data, did a post‐hoc analysis in which they added predictors of how related the verb and noun were to their figurative meanings. The authors found that verb relatedness made reading times faster, whereas they did not find an effect of noun relatedness. These findings suggest that the verb and noun in verb‐object idioms are processed differently.

The studies discussed above looked at idiom comprehension; we now briefly survey work that has looked at production. Consistent with the results from comprehension studies, the consensus from work on production is that idioms are spoken faster than literal controls (Lovseth et al., 2011; Siyanova‐Chanturia & Lin, 2018; Van Lancker et al., 1981). In an early study, Van Lancker et al. (1981) compared the prosody of idiomatic and literal interpretations of the same verb‐object phrase. Two experiments were run, one where participants were explicitly instructed to read the sentence with a particular interpretation, and another in which participants were instructed to read the sentences naturally and were not told given a particular interpretation. The authors found that idioms were spoken faster in the experiment where participants were told to produce a particular meaning, but that there was no significant difference in the experiment where the sentences were read naturally. Lovseth et al. (2011) looked at natural productions of verb‐object phrases that have both idiomatic and literal interpretations and *did* find that idiomatic phrases had shorter durations. Additionally, all of the differences the authors found between the idiomatic and literal conditions manifested on the object noun. They did not find a significant effect of decomposability on duration.^2^

A significant way in which our studies differ from previous work is that we compare idioms to syntactically matched phrases that do not have possible idiomatic interpretations, whereas Van Lancker et al. (1981), Bélanger et al. (2009), and Lovseth et al. (2011) compared idiomatic and literal interpretations of the same phrase. Our reasoning for this choice is to avoid any confounding effects of accessing the interpretation that is not intended in a particular trial. In addition, our production study is substantially larger than the majority of previous studies in this area.

## 3 A fine‐grained measure of compositionality

The objective of the current work is to determine the extent to which surprisal can explain the idiom processing advantage. In order to test this, we include as an additional predictor in our studies a measure that captures the compositionality of a phrase, since compositionality has been frequently hypothesized to play a role in idiom processing. In this section, we discuss our choice of compositionality measure.

We begin by noting that within linguistics, compositionality has traditionally been defined as a homomorphism between linguistic structure and meaning (Montague, 1970). Under this definition, compositionality is a property of a system as a whole; it is, therefore, incoherent to talk about individual utterances within that system as differing in their compositionality (Pagin & Westerståhl, 2010). Furthermore, compositionality under traditional formalisms is binary—a system is either compositional or not, with no room for degrees of compositionality. Yet, in the literature on idiom processing (and beyond), compositionality is commonly described as an utterance‐specific and gradient property.^3^ This mismatch between the phenomenon people want to describe and its traditional formalization has led to a proliferation of gradient compositionality measures within the idiom processing literature, which have been developed separately from the theoretical compositionality literature. Importantly, these measures tend to conflate a number of different issues. For example, the most common such measure is *decomposability* (discussed in Section 2), which has yielded conflicting results in attempts to explain the idiom processing advantage. We suspect that a possible reason for these conflicting results is that the measure is not precise enough, as it does not capture the fact that the individual words in an idiom can differ in their how transparent their meanings are (e.g., *spill* is closer to “reveal” than *beans* is to “information/secret”).

We adopt a theory of compositionality, laid out in Socolof (2024), wherein an utterance is fully compositional if its meaning is fully predictable given the meanings of its subparts across contexts beyond the utterance in question. Furthermore, utterances can differ in their degree of compositionality along a gradient scale, depending on the extent to which the subparts contribute independent canonical meanings. Under this theory, it is coherent to talk about the degree of compositionality associated with a particular word's contribution to a larger utterance. In the current study, we opt for a measure that was built upon these theoretical underpinnings, and which was developed independently from work on idiom processing. The measure is called *conventionality* and was proposed in Socolof, Cheung, Wagner, and O'Donnell (2022); it uses large language model‐generated contextual word embeddings to estimate the distance between a word's meaning in a particular context and its average meaning across other contexts. Crucially, the measure is localized to individual words, providing a fine‐grained quantity that gets at an intuitive notion of compositionality. Socolof et al. (2022) showed that words in idioms have lower conventionality scores than the same words in syntactically matched literal phrases; in the current study, we found this to be true for our dataset, for both verbs and nouns.

## 4 Experiment 1: Comprehension

The idiom processing advantage has been robustly established in the literature on sentence comprehension; we investigated whether surprisal could account for this effect. For this study, we carried out an online self‐paced reading experiment in which participants read sentences containing idiomatic verb‐object as well as sentences containing literal verb‐object phrases. The core predictions were that we would observe an idiom processing advantage in the form of shorter reading times for idioms than for literal phrases, and that surprisal would largely explain the difference in reading times.

### 4.1 Participants

Participants were monolingual adult native speakers of North American English recruited on Prolific (www.prolific.com). There were 75 participants (37 female, 32 male, 6 other), ranging in age from 21 to 71 (mean 37.9, SD 12.73). Participants gave written informed consent and were compensated at a rate of $15/h.

### 4.2 Materials

Each stimulus was a sentence containing a verb‐object phrase. There were three conditions: one where the verb‐object phrase was an idiom, one where the verb‐object phrase was nonidiomatic and matched the verb of the idiom, and one where the verb‐object phrase was nonidiomatic and matched the noun of the idiom. The words in the verb‐object phrases had the same number of syllables across the three conditions. An example item set containing the three conditions is given in (4.2).

(1)
  1. (Idiom) Their policy on childcare has **struck a chord** with family voters.
  2. (Verb match) The army **struck the town** without warning.
  3. (Noun match) The pianist **played a chord** to open the concert.

There were 17 idioms in the experiment, each of which had five item sets, for a total of 255 stimuli. In each stimulus, the verb‐object phrase occurred sentence‐medially, followed by an adjunct phrase. Every idiom in the experiment had associated conventionality scores. The stimuli were split up into five subexperiments, each containing one item set per idiom (so 51 stimuli). We recruited 15 participants for each subexperiment, such that those participants only saw the items in that subexperiment. Each participant saw all of the items in a single subexperiment, presented in randomized order.

### 4.3 Procedures

Items were presented one word at a time in a self‐paced moving window paradigm (Just, Carpenter, & Woolley, 1982) with the PCIbex Farm software (Zehr & Schwarz, 2018). Each trial began with a screen where a row of dashes masked all words in the sentence. Participants pressed the space bar to reveal the first word and each subsequent word. When a new word was revealed, the previous word was remasked. Half of the items were followed by a true/false comprehension question, balanced by condition and by true/false answer.

### 4.4 Analysis

The regions of analysis in our mixed‐effects models were the words in the verb‐object target phrases (the verb, noun, and potentially a determiner), and the subsequent word (the spillover region). The details of the models are given below.

### 4.5 Surprisal and conventionality values

For each stimulus sentence in the experiment, surprisal values were estimated using a language model, GPT‐3, for each word in the target phrase given the preceding words. Conventionality scores for the target verb and noun in each sentence were computed using the procedure described in Socolof et al. (2022), which uses the BERT language model (Devlin, Chang, Lee, & Toutanova, 2019) to measure the distance between a word's embedding in a particular context and the word's average embedding across other contexts. The conventionality of a word in a particular phrase is computed by first taking the average embedding for the word across sentences not containing the phrase. Let O be a set of instances $w_{1},w_{2},\text{…},w_{n}$ of a particular word used in contexts other than the context of the phrase in question. Each instance has an embedding $u_{w_{1}},u_{w_{2}},\text{…},u_{w_{n}}$. The average embedding for the word among these sentences is:

(2) $$\mu_{O}=\frac{1}{n}\sum_{i=1}^{n}u_{w_{i}}.$$

This quantity is taken as an estimate of the conventional meaning of the word. The conventionality score is the negative of the average distance between $\mu_{O}$ and the embeddings for uses of the word across instances of the phrase in question:

(3) $$conv\left(phrase\right)=-\frac{1}{m}\sum_{i=1}^{m}{\left\|\frac{T_{i}-\mu_{O}}{\sigma_{O}}\right\|}_{2},$$

where T is the embedding corresponding to a particular use of the word in the phrase in question, and $\sigma_{O}$ is the component‐wise standard deviation of the set of embeddings $u_{w_{i}}$, and m is the number of sentences in which the particular phrase is used.

### 4.6 Results

Mean accuracy on the comprehension questions was 89%. We first present comparisons of the surprisal and conventionality values across conditions, then we report mixed‐effects analyses.

**Fig. 1:**
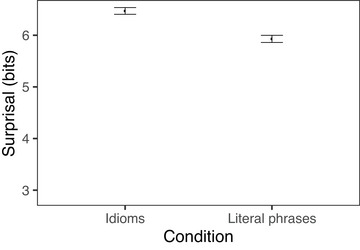
Surprisal of verb in verb‐object idioms and verb‐matched literal phrases. Error bars indicate 95% bootstrapped confidence intervals.

**Fig. 2:**
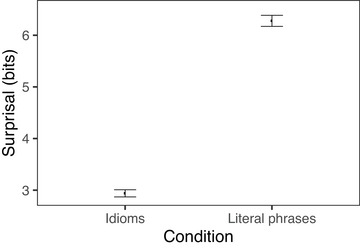
Surprisal of noun in verb‐object idioms and noun‐matched literal phrases. Error bars indicate 95% bootstrapped confidence intervals.

Looking at surprisal, we find that idiomatic and literal phrases differ significantly in overall surprisal (*t* = −20, *p* < .001), with idiomatic phrases having lower surprisal (mean = 9.41) than literal phrases (mean = 12). This difference was driven primarily by the noun, with the nouns in idiomatic verb‐object phrases having an average surprisal of 2.94 bits, whereas the nouns in literal phrases had an average surprisal of 6.28 bits (*t* = −26, *p* < .001). There was a much smaller difference between verbs across conditions, and the effect actually went in the opposite direction, with idiomatic verbs having higher surprisal (mean = 6.47) than literal verbs (mean = 5.93; *t* = 6, *p* < .001).^4^ Figs. 1 and 2 show the differences in surprisal between idioms and literal phrases for the verb and noun, respectively.

**Fig. 3:**
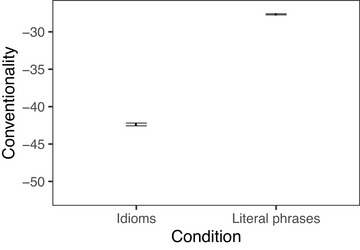
Conventionality of verb in verb‐object idioms and verb‐matched literal phrases. Error bars indicate 95% bootstrapped confidence intervals.

**Fig. 4:**
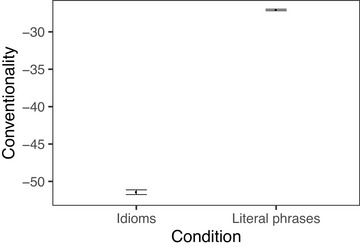
Conventionality of noun in verb‐object idioms and noun‐matched literal phrases. Error bars indicate 95% bootstrapped confidence intervals.

We now turn to the results for the conventionality measure. Conventionality, when computed for idioms, is meant to capture how different the meaning of a word is in idiomatic contexts from the word's literal meaning, and is measured for individual words rather than full phrases. For both the verbs (Fig. 3) and nouns (Fig. 4) in our dataset, those appearing in idioms were less conventional than those in literal phrases, as expected. Idiomatic verbs had a mean conventionality score of −42.4 compared to −27.7 for literal verbs (*t* = −73, *p* < .001), and idiomatic nouns had a mean of −51.4 compared to −27.1 for literal nouns (*t* = −74, *p* < .001).

**Fig. 5:**
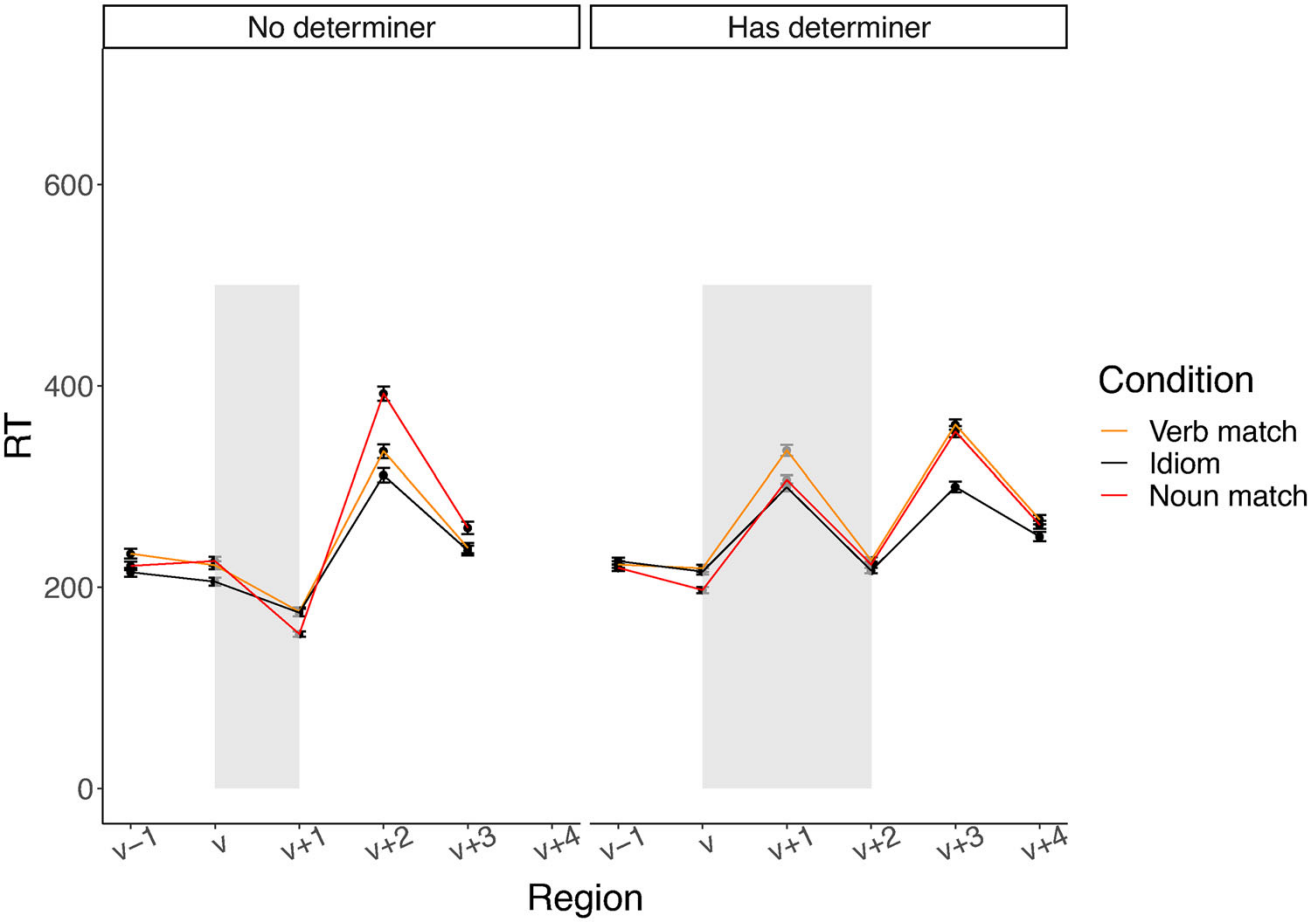
Self‐paced reading reaction times in milliseconds for the region spanning one word before the target phrase to two words after, separated by whether there is a determiner between the verb and noun. The target phrase is shaded gray.

Fig. 5 shows mean reading times for the three conditions spanning the word before the idiom to two words after the idiom. A visual inspection suggests that idioms are read more quickly than literal phrases, with the difference in reading time occurring primarily in the spillover region. We carried out separate mixed‐effects analyses with RT as the dependent variable for four different sentence positions: the verb, the determiner (for the subset of trials that had one), the noun, and the word following the noun (the spillover region). Raw RTs (as opposed to transformed RTs) were used because word surprisal effects are expected to be linear in raw RTs (Burchill & Jaeger, 2024; Smith & Levy, 2013). The models were fit using the lmerTest (Kuznetsova, Brockhoff, & Christensen, 2017) package in R (R Core Team, 2017). The verb and determiner models were used to investigate the effect of the verb, so for those models, we included as fixed effects verb surprisal, verb conventionality, and their interaction. For the noun and spillover models, which we used to investigate the effect of the noun and potentially spillover effects of the verb, we included noun surprisal, noun conventionality, their interaction, as well as verb surprisal and the interaction between verb and noun surprisal. The continuous variables were rescaled by centering and dividing by two standard deviations. All models were first fit using maximal random effect structure, and in cases of nonconvergence, random slopes were removed starting with those that accounted for the least variance. We found a significant effect of verb surprisal in the determiner model (reported in Table 1), and of noun surprisal in the spillover model (reported in Table 2); there were no significant effects in the verb or noun models. Our finding that reading time effects in idiom processing occur in the spillover region is consistent with previous self‐paced reading studies on idioms (Beck & Weber, 2020; Holsinger & Kaiser, 2013). 

**Table 1:** Model results table with determiner reaction time as the dependent variable. Asterisks indicate significance using a threshold of *p* = 0.05.

Coefficient	$\hat{\beta }$	$SE(\hat{\beta })$	t	p
Intercept	0.255	0.037	6.99	<.001*
VerbSurprisal	0.060	0.021	2.93	.005*
VerbConventionality	0.035	0.020	1.75	.085
VerbSurprisal:VerbConventionality	−0.077	0.044	−1.74	.083

*Note*. *n* = 1155.

**Table 2:** Model results table with reaction time of the word after the noun as the dependent variable. Asterisks indicate significance using a threshold of *p* = 0.05.

Coefficient	$\hat{\beta }$	$SE(\hat{\beta })$	t	p
Intercept	0.005	0.045	0.12	.906
NounSurprisal	0.063	0.023	2.77	.007*
NounConventionality	0.010	0.021	0.46	.647
VerbSurprisal	0.017	0.022	0.78	.439
NounSurprisal:NounConventionality	0.028	0.040	0.72	.476
NounSurprisal:VerbSurprisal	0.009	0.043	0.21	.838

*Note*. *n* = 1805.

## 5 Experiment 2: Production

Given that most work on idioms in psycholinguistics has focused on comprehension, we also conducted a production study in order to see whether we observe a similar advantage as in comprehension, and if so, whether surprisal can explain the data. To do this, we carried out an online experiment in which participants read aloud individual sentences, some of which contained idioms and some of which contained syntactically matched nonidiomatic phrases. The experiment was created using the prosodylab‐Experimenter (Wagner, 2021), whose functionality is built upon jsPsych (De Leeuw, 2015).

### 5.1 Participants

We recruited 134 adult native speakers of North American English on Amazon Mechanical Turk. Of these, 44 were excluded for not being native speakers or for unintelligible recordings, leaving 90 participants (39 female, 49 male, 2 other) who ranged in age from 25 to 74 (mean 35, SD 10.9). Participants gave written informed consent and were compensated at a rate of $15/h.

### 5.2 Materials

The same stimuli were used as in the comprehension experiment, except that we doubled the number of item sets for each idiom such that half the items had the phrase of interest in sentence‐final position and the other half in sentence‐medial position (followed by an adjunct phrase). We created novel sentences for the sentence‐final items. There were 17 idioms in the experiment, each of which had 10 item sets (each consisting of the three conditions), for a total of 510 stimuli. Every idiom in the experiment had associated conventionality scores. The item sets were split into 10 subexperiments, each containing one item set per idiom (so 51 stimuli). As in the comprehension experiment, we recruited 15 participants for each subexperiment. Each participant saw all of the items in a single subexperiment, presented in randomized order.

### 5.3 Procedures

Participants were instructed to complete the experiment in a quiet room. They first performed a microphone check, during which they recorded themselves saying a sentence out loud with their computer microphone and listened back to the recording. At the beginning of the experiment, participants read an instruction screen, where they were told that they would be presented with one sentence at a time, and that they should first read the sentence silently to themselves, then click the record button and read the sentence out loud, and finally click the button to stop recording. They were told to read the sentences as naturally as possible. Once consent was given and the experiment began, the trials appeared in full‐screen mode. For each trial, the sentence to be read aloud was written in boldface in the center of the screen. Below the sentence was text that read, “Read the sentence silently to yourself. When you're ready, click the button below to record.” Below this was a button that read, “Click here to start recording.” After the participant clicked this button, they were taken to a screen with the same sentence in bold in the middle of the page, with the text, “Please speak now,” as well as a button reading, “Click here when you're done recording.” Clicking this button moved them to the next trial. Participants could choose to press the space bar instead of clicking the button if they wished.

### 5.4 Acoustic analysis

Each sentence recording was force aligned using the Montreal Forced Aligner (McAuliffe, Socolof, Mihuc, Wagner, & Sonderegger, 2017). There were 4860 individual recordings from the participants who followed instructions on the practice sentence. Of these recordings, 4794 were successfully force‐aligned to the sentence in question. Acoustic measures of duration, pitch (mean, max, and min), and intensity (mean, max, and min) were then extracted for the words of interest (the verb and noun in the verb‐object phrase, plus the first nonfunction word in the sentence and the sentence‐final word for those stimuli where the verb‐object phrase was not sentence‐final).

### 5.5 Surprisal and conventionality values

The same procedure was used as in the comprehension experiment.

### 5.6 Results

We observed significant effects for duration, but none for pitch or intensity. We note that of these metrics, duration effects have been the most robustly found in other work comparing idiomatic and literal phrases (Bélanger et al., 2009; Lovseth et al., 2011; Van Lancker et al., 1981). Pitch effects have yielded mixed findings; they were found in Van Lancker et al. (1981) and Lovseth et al. (2011), but not in Bélanger et al. (2009).

**Fig. 6:**
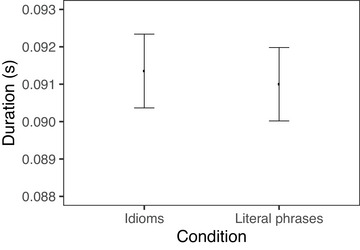
Duration of verb in verb‐object idioms and verb‐matched literal phrases. Error bars indicate 95% bootstrapped confidence intervals.

**Fig. 7:**
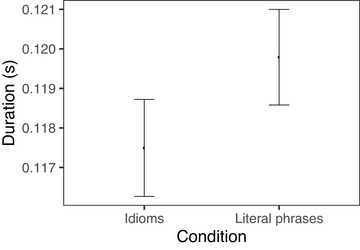
Duration of noun in verb‐object idioms and noun‐matched literal phrases. Error bars indicate 95% bootstrapped confidence intervals.

We first report the difference in duration between idiomatic and nonidiomatic phrases as a whole, then we report statistical models investigating the effects of surprisal and conventionality on verbs and nouns separately. When reporting model results, we use average phone duration as our duration measure. We find that idiomatic verbs and nouns taken together were produced with significantly shorter durations than literal phrases, in line with most of the existing literature. This effect is notably quite small; in our experiment, there was an average difference of 9 ms between idioms and literal phrases, which was 1.2% of the duration of the literal phrases overall. Figs. 6 and 7 show verb and noun durations, respectively, for idioms versus literal phrases. We observe that the noun is shortened in the idiom condition, whereas there is no obvious difference in verb duration between idioms and literal phrases.

To investigate the effects of surprisal and conventionality on the spoken duration of idioms versus literal verb‐object phrases, we ran separate models for the verb and the noun. We begin with the model for verb duration. We fit a linear mixed model with verb duration as the dependent variable and verb surprisal and verb conventionality score as main predictors, as well as the surprisal of the preceding word (to account for spillover effects). Model comparison indicated that including the interaction of verb surprisal and verb conventionality improved the model. We also included random effects for participant, target phrase, and item, using a backward selection procedure to arrive at a model that was maximal up to convergence. The model was fit using the formula

(2) verbDuration ∼ verbSurprisal + verbConventionality + previousWordSurprisal + verbSurprisal:verbConventionality + MedialOrFinal + (1 + verbSurprisal + verbSurprisal:verbConventionality | participant) + (1 + previousWordSurprisal + MedialOrFinal | idiom) + (1 | item).

**Fig. 8:**
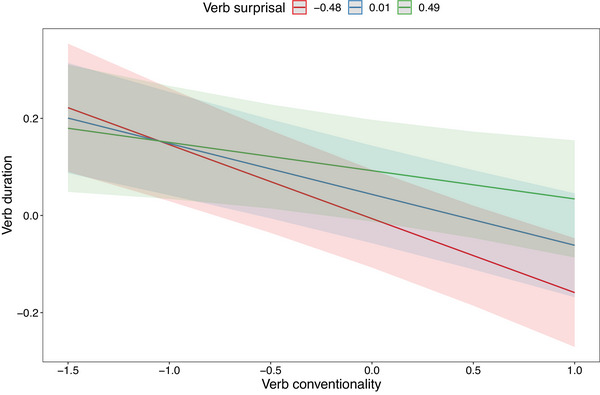
Interaction of verb surprisal and verb conventionality on verb duration.

The continuous variables were rescaled by centering and dividing by two standard deviations. Results are shown in Table 3. We find that verb surprisal, verb conventionality, sentence position, and the interaction between verb surprisal and verb conventionality have significant effects on verb duration. The main effects indicate that (1) surprising verbs are spoken more slowly than predictable verbs, and (2) verbs with unconventional meanings in context are spoken more slowly than more literal usages of verbs. As for the interaction of surprisal and conventionality, we find that for surprising verbs, conventionality has no significant correlation with duration, whereas for predictable verbs, greater conventionality is correlated with shorter duration. The interaction is shown in Fig. 8, where we see that at higher levels of verb conventionality, there is a greater difference in duration based on verb surprisal. Note that verb surprisal and verb conventionality are weakly negatively correlated (−0.133, *p* = .02).

**Table 3:** Model results table with verb duration as the dependent variable. Asterisks indicate significance using a threshold of *p* = 0.05.

Coefficient	$\hat{\beta }$	$SE(\hat{\beta })$	t	p
Intercept	0.071	0.052	1.36	.181
VerbSurprisal	0.104	0.022	4.68	<.001*
VerbConventionality	−0.107	0.018	−5.81	<.001*
PreviousWordSurprisal	0.090	0.047	1.91	.072
MedialOrFinal	−0.130	0.051	−2.55	.012*
VerbSurprisal:VerbConventionality	0.103	0.045	2.29	.023*

*Note*. *n* = 2611.

We fit a similar model for noun duration. Since the noun is encountered after the verb, we included verb‐related predictors, choosing from among models using a nested likelihood‐ratio test. We found that the best model did not include verb conventionality, and like for the verb model, the structure was maximal up to convergence. Our model is given by the formula

(3) nounDuration ∼ nounSurprisal + nounConventionality + nounSurprisal:nounConventionality + verbSurprisal + nounSurprisal:verbSurprisal + MedialOrFinal + (1 + nounSurprisal + verbSurprisal + nounSurprisal:nounConventionality | participant) + (1 + verbSurprisal + MedialOrFinal | idiom) + (1 | item).

As in the verb model, all continuous predictors were rescaled by centering and dividing by two standard deviations. Results are shown in Table 4.

**Table 4:** Model results table with noun duration as the dependent variable. Asterisks indicate significance using a threshold of *p* = 0.05.

Coefficient	$\hat{\beta }$	$SE(\hat{\beta })$	t	p
Intercept	0.205	0.083	2.46	.021*
NounSurprisal	0.171	0.024	7.15	<.001*
NounConventionality	0.005	0.021	0.22	.823
VerbSurprisal	0.112	0.050	2.24	.039*
MedialOrFinal	−0.339	0.066	−5.18	<.001*
NounSurprisal:NounConventionality	−0.028	0.041	−0.69	.489
NounSurprisal:VerbSurprisal	−0.131	0.038	−3.46	<.001*

*Note*. *n* = 2687.

**Fig. 9:**
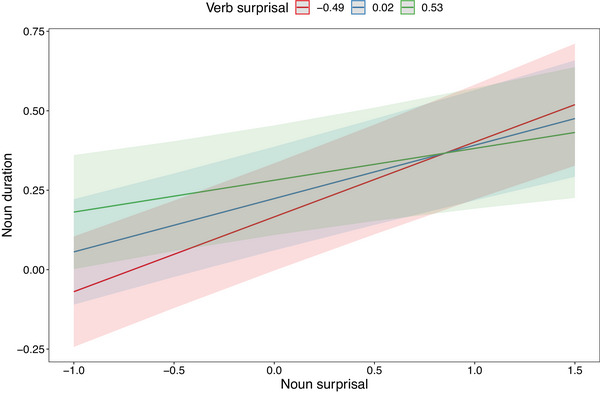
Interaction of noun surprisal and verb surprisal on noun duration.

We find that noun surprisal has a large effect on the duration of the noun, with more surprising nouns being pronounced more slowly. Unlike for verbs, we do not find any additional significant additional effect of noun conventionality on noun duration, and there is no significant interaction between noun surprisal and noun conventionality. We find that the surprisal of the verb is positively correlated with the duration of the noun, and that there is an interaction between noun surprisal and verb surprisal, whereby the longer duration associated with surprising nouns is more pronounced when the preceding verb is highly predictable. The interaction is plotted in Fig. 9, which shows that when noun surprisal is low, there is a greater effect of verb surprisal on noun duration. Finally, we see a large effect of sentence‐final lengthening on the noun, which we take to be orthogonal to to the research questions at hand. Among nouns, the correlation between surprisal and conventionality is 0.41 (*p* < .001).

## 6 Discussion

The results of our comprehension study paint a straightforward picture of the role of surprisal in idiom processing; we see a significant effect of a word's surprisal on RT, with idioms having lower surprisal and shorter RTs than literal phrases, and no significant effect of conventionality. These results provide evidence for surprisal as the relevant variable in explaining the idiom processing advantage in comprehension, rather than measures related to compositionality. With the production study, we sought to test whether the idiom advantage also manifested in production, and if so, whether it could also be explained by surprisal. It is not necessarily obvious that surprisal should play a role in production; unlike in the comprehension study, participants in the production study saw each sentence in its entirety before producing it, so they should not have experienced incremental updates to their beliefs about subsequent words in the course of producing the sentence. Any effect of surprisal on production, therefore, requires explanation.

In the production study, our first finding is that verb‐object idioms are spoken more quickly on average than literal verb‐object phrases, which is consistent with the consensus of previous work. The overall shorter duration of idioms is due to a difference on the nouns; there is no significant difference between the duration of verbs across the two groups. We further find that the shorter duration of idioms is driven largely by the low surprisals associated with idiomatic nouns. The fact that the bulk of the idiom advantage can be explained by surprisal without making reference to compositionality sheds light on why studies that probed for compositionality without taking surprisal into account have reported mixed results.

What do our results suggest about the mechanisms underlying idiom production? If the production task were taken to reflect incremental processing, then we might explain the results by saying that when one has already encountered a verb that strongly predicts a particular idiom, then seeing the noun that completes that idiom simply confirms one's expectations. However, given that participants read each sentence prior to producing it, we cannot assume a straightforward incremental processing story. We offer two alternative possibilities for why production data might be explained by surprisal. The first is that production may reflect audience design considerations—specifically, speakers using a communication strategy that modulates word duration based on surprisal for the comprehender's benefit (Arnold, 2008; Brennan & Williams, 1995; Clark & Murphy, 1982; Lindblom, 1990). If we think of production time in terms of a speaker providing information to a listener, then a longer duration at a certain word may indicate that word is highly informative and particular attention should be paid to it. The second possibility is that words that have low surprisal in context may facilitate planning during the production process (Bock & Warren, 1985), and that this manifests as shorter duration on those low‐surprisal words. In order to distinguish between these and other potential factors, further investigation into production mechanisms is necessary.

While we see similar patterns across the comprehension and production data, there is an important difference: unlike in the comprehension study, we observe in the production study that conventionality plays a small role beyond that of surprisal. The locus of the idiom advantage is on the noun, yet, we nevertheless find, in the verb model, effects of verb conventionality and the interaction between verb conventionality and verb surprisal. Why do we see this residue of conventionality affecting verb duration? If surprisal were a true causal bottleneck, then we would not expect to see any residue, so the fact that we do provides suggestive evidence against the causal bottleneck theory, or else evidence that it at least does not apply to the mechanisms of production. A potentially important factor in explaining the lack of conventionality effects in comprehension is that conventionality is computed with reference to the full surrounding context of a word, since it seeks to capture the extent to which a word is used in a canonical way in its syntactic position within a particular sentence. During the task of incremental sentence comprehension, one may not immediately be able to tell if a word is being used in a conventional way, which could explain the lack of conventionality effect. In production, on the other hand, conventionality is known during the planning process and, therefore, can more straightforwardly play a role in production patterns.

## 7 Conclusion

We investigated differences in RT and spoken duration between verb‐object idioms and syntactically matched nonidioms, and the effects of surprisal and conventionality in accounting for these differences. We found evidence of the idiom processing advantage in both comprehension and production, with a significant effect of surprisal in both studies. In production, we also found that surprisal and conventionality interact to affect verb duration, but that noun duration primarily reflects surprisal. One question raised by these results is whether a similar pattern exists in phrases with syntactic structures other than verb‐object. Socolof et al. (2022) found that across syntactic structures, head words tend to have more conventional meanings than their dependents, so it would be interesting to investigate the processing of idioms where the syntactic head of the phrase comes after the dependent, as this would help tease apart effects of left‐to‐right processing versus head‐dependent asymmetries on processing. For example, what does the pattern look like for adjective‐noun idioms such as *cold feet* and *sour grapes*? Such studies, informed by the role of surprisal, would help paint a fuller picture of idiom processing effects (Supplementary material).

## Ethics approval

This study was carried out with the approval from the McGill Research Ethics Board protocols #401‐0409/#342‐0118.

## Supporting information

Data S1[Supplementary-material cogs70085-supl-0001]

## Data Availability

All codes and anonymized data will be made available.

## Notes

1 However, see Kyriacou, Conklin, and Thompson (2021) for a result that idioms are processed more slowly. The authors suggest that this effect may have been due to the cost of disambiguating between the idiomatic and literal interpretation of a phrase when preceded by a biasing context.

2 See Bélanger, Baum, & Titone (2009) for a result where idiomatic nouns were produced more slowly than literal nouns.

3 For further discussion of this issue, see Socolof (2024).

4 Note that considering verb and noun surprisal together reveals that idiomatic phrases have less surprisal overall than matched literal phrases.
