# Supplementary material for: The Idiom Processing Advantage is Explained By Surprisal
Source: Cogn Sci. 2025 Jul 20;49(7):e70085. doi: 10.1111/cogs.70085 (PMC12276763; doi:10.1111/cogs.70085)
Supplement: Supplementary file 1 — Data S1 [file COGS-49-e70085-s001.pdf]

item	condition	phrase	sentence
1	1	spill the beans	Everyone is worried that the politician will spill the beans about the fundraiser.
1	2	spill the beans	"In a fit of rage, the toddler decided to spill the juice all over the floor."
1	3	spill the beans	The farmer woke up early to plant the beans in the bean-field.
2	1	call the shots	"In this country, the president is the one to call the shots about military operations."
2	2	call the shots	"Whenever I feel sick, I call my mom to help me out."
2	3	call the shots	The prisoner wants a chance to call his son to wish him a happy birthday.
3	1	clear the air	I asked my supervisor to meet so I could clear the air about my recent absences.
3	2	clear the air	The barman cleared the bar to make space for a new guest.
3	3	clear the air	I love to breathe the air in the mountains.
4	1	have a ball	I used to hate exercise but now I have a ball at the gym.
4	2	have a ball	I can't wait to have a cat in my new apartment.
4	3	have a ball	My dog loves when I throw a ball in the backyard.
5	1	get the sack	She got the sack for always being late.
5	2	get the sack	I stopped by the bakery and got the cake for my dad.
5	3	get the sack	"Every day, the farmer fills the sacks with grain."
6	1	bite the dust	Many young people’s hopes and ambitions bit the dust during the recession.
6	2	bite the dust	It was Eve who bit the fruit in the garden of Eden.
6	3	bite the dust	The hotel cleaner sweeps the dust to keep the rooms impeccably clean.
7	1	break the mold	"I come from a long line of doctors, but I wanted to break the mold by becoming a singer."
7	2	break the mold	Harry broke a vase in the living room.
7	3	break the mold	You must make a mold before filling it with concrete.
8	1	lose ground	We cannot waste time while our soldiers lose ground on the battlefield.
8	2	lose ground	It is very common for people to lose keys on their way to work.
8	3	lose ground	The workers cleared ground for the new garden.
9	1	make waves	The mayor's announcement made waves

in the neighborhood.

9 2 make waves I hear waves from my bedroom.

9 3 make waves I love to catch waves in the sea.

10 1 cut corners It is certainly not a sensible move to cut corners with national security.

10 2 cut corners Kids love to cut circles into cardboard paper.

10 3 cut corners I am always scared of running into people when I turn corners in the city.

11 1 raise hell Chester was still in the party and ever ready to raise hell in the house.

11 2 raise hell It is not always easy to raise kids in this country.

11 3 raise hell Orpheus turned around and saw hell.

12 1 pull strings I may be able to pull strings for you.

12 2 pull strings My toddler unfornately likes to grab and pull hair from our dog.

12 3 pull strings The seamstress tied strings into a nice bow.

13 1 mean business It sounded like Mom means business with her renovation project.

13 2 mean business This necklace means nothing to me.

13 3 mean business The company is afraid it will lose business by being too political.

14 1 turn tail The fox quickly turned tail after seing the hunter.

14 2 turn tail Movie theaters turn chairs towards the screen.

14 3 turn tail "For this game, we have to pin tails on a donkey."

15 1 rock the boat Don't rock the boat without my permission.

15 2 rock the boat This dad likes to rock his child to sleep.

15 3 rock the boat Big waves flipped the boat in the open sea.

16 1 lead the field Americans continue to lead the field when it comes to child actors.

16 2 lead the field The wolf led the kids into the forest.

16 3 lead the field The festival attendees were asked to clear the field after the show.

17 1 strike a chord Their policy on childcare has struck a chord with family voters.

17 2 strike a chord The army struck the town without warning.

17 3 strike a chord The pianist played a chord to open the concert.

18 1 run the show They made it clear who runs the show

around the office.
18 2 run the show The billionaire runs a mine in South Africa.
18 3 run the show I used to watch that show with my mom.
0 0 catchtrial The dog dug in the yard for a bone.
0 0 catchtrial Exactly nine children were given extra time on their tests.
